# Injectable Porous Microspheres Loaded With Biomimetic Preconditioned Bone Marrow Mesenchymal Stem Cell‐Derived Exosomes for Vascularized Bone Regeneration

**DOI:** 10.1002/advs.74987

**Published:** 2026-03-24

**Authors:** Lijun Li, Hao Zhang, Lingtong Sun, Yingfeng Su, Yang Xu, Jian Huang, Jingchao Wen, Jinjin Zhu, Jianjun Ma, Wenbin Xu

**Affiliations:** ^1^ Department of Orthopaedic Surgery Sir Run Run Shaw Hospital Zhejiang University School of Medicine & Zhejiang Key Laboratory of Mechanism Research and Precision Repair of Orthopaedic Trauma and Aging Diseases Hangzhou Zhejiang China; ^2^ College of Pharmaceutical Sciences Zhejiang Chinese Medical University Hangzhou China; ^3^ Hangzhou Xixi Hospital Affiliated to Zhejiang Chinese Medical University Hangzhou Zhejiang China; ^4^ Department of Thoracic Surgery Zhejiang Cancer Hospital Hangzhou Zhejiang China; ^5^ Department of Ultrasound Sir Run Run Shaw Hospital Zhejiang University School of Medicine Hangzhou Zhejiang China; ^6^ Department of Orthopaedic Surgery the Fourth Affiliated Hospital of School of Medicine International Institutes of Medicine, and International School of Medicine International Institutes of Medicine Zhejiang University Yiwu China

**Keywords:** angiogenesis, biomimetic preconditioning, bone regeneration, exosomes, injectable porous microspheres, tissue engineering

## Abstract

Critical‐sized bone defects remain a highly challenging clinical problem due to insufficient intrinsic self‐healing capacity. Bone marrow mesenchymal stem cell (BMSC)‐derived exosomes have emerged as promising cell‐free therapeutic candidates for bone regeneration, owing to their paracrine effects in regulating bone regeneration‐related processes. However, enhancing exosome bioactivity via biomimetic preconditioning and developing efficient delivery vectors remain key bottlenecks in this field. Herein, we developed a synergistic bone regenerative system composed of biomimetic preconditioned BMSC‐derived exosomes (BioPre‐Exos) and injectable porous polydopamine (PDA)‐modified gelatin methacryloyl (GelMA) microspheres. The biomimetic preconditioning strategy adopted 3% hypoxia combined with 3D GelMA microsphere culture, mimicking the bone marrow microenvironment to regulate BMSC functions and significantly boost exosome bioactivity. Functional experiments verified that BioPre‐Exos robustly promoted BMSC migration, osteogenic differentiation, angiogenesis, and macrophage polarization toward an anti‐inflammatory phenotype in vitro. Furthermore, in a rat femoral condyle defect model, the composite system markedly improved neovascularization density and bone volume fraction, thus achieving efficient vascularized bone regeneration. These findings indicate that this cell‐free biomimetic synergistic delivery system holds great application potential in the clinical treatment of bone defects.

## Graphical abstract

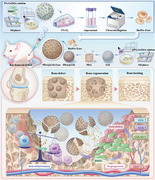

## 1 Introduction

Bone defects, primarily caused by trauma, infection, and tumor resection, represent prevalent and challenging problems in clinical orthopedics [1, 2]. Among these, critical‐sized bone defects often result in persistent nonunion, motor dysfunction, and a significant decline in patients' quality of life due to insufficient intrinsic regenerative capacity [3, 4]. To address this clinical need, tissue engineering strategies based on bioactive scaffolds have emerged as a highly promising therapeutic paradigm for bone regeneration [5, 6, 7]. Compared with mesenchymal stem cell (MSC) transplantation, exosome‐mediated cell‐free therapy exhibits distinct advantages, such as low immunogenicity and no tumorigenic risk, thereby demonstrating substantial therapeutic potential in bone defect repair [8, 9]. Notably, hypoxic conditions in the bone marrow microenvironment are core physiological factors regulating the functions of bone marrow mesenchymal stem cells (BMSCs) and the secretory properties of exosomes [10]. However, current research faces a critical bottleneck where the majority of therapeutic exosomes derived from BMSCs are obtained via 2D monolayer culture combined with single‐factor preconditioning [11, 12, 13, 14, 15]. Such systems fail to recapitulate key characteristics of the bone marrow microenvironment, including hypoxic gradients and 3D cell‐cell/cell‐matrix interactions [9]. Although few studies [16, 17] have attempted to harvest exosomes by seeding BMSCs on hydrogel film surfaces, these systems are essentially 2D adherent models that cannot synergistically recapitulate the 3D spatial structure and hypoxic microenvironment of the bone marrow, leading to insufficiencies in the osteogenic and angiogenic regulatory activities of the resulting exosomes. Moreover, conventional hydrogel‐based exosome delivery systems have inherent drawbacks as they exhibit high rigidity and poor injectability, which are not conducive to minimally invasive administration and cannot adapt to the irregular contours of bone defects [8, 11, 18]. Additionally, their release depends on passive diffusion, which tends to cause burst release of exosomes and severely compromises long‐term therapeutic efficacy [15, 18]. Thus, the development of biomimetic carriers for efficient delivery of functionally enhanced exosomes represents a promising research direction in bone defect repair.

**FIGURE 1:**
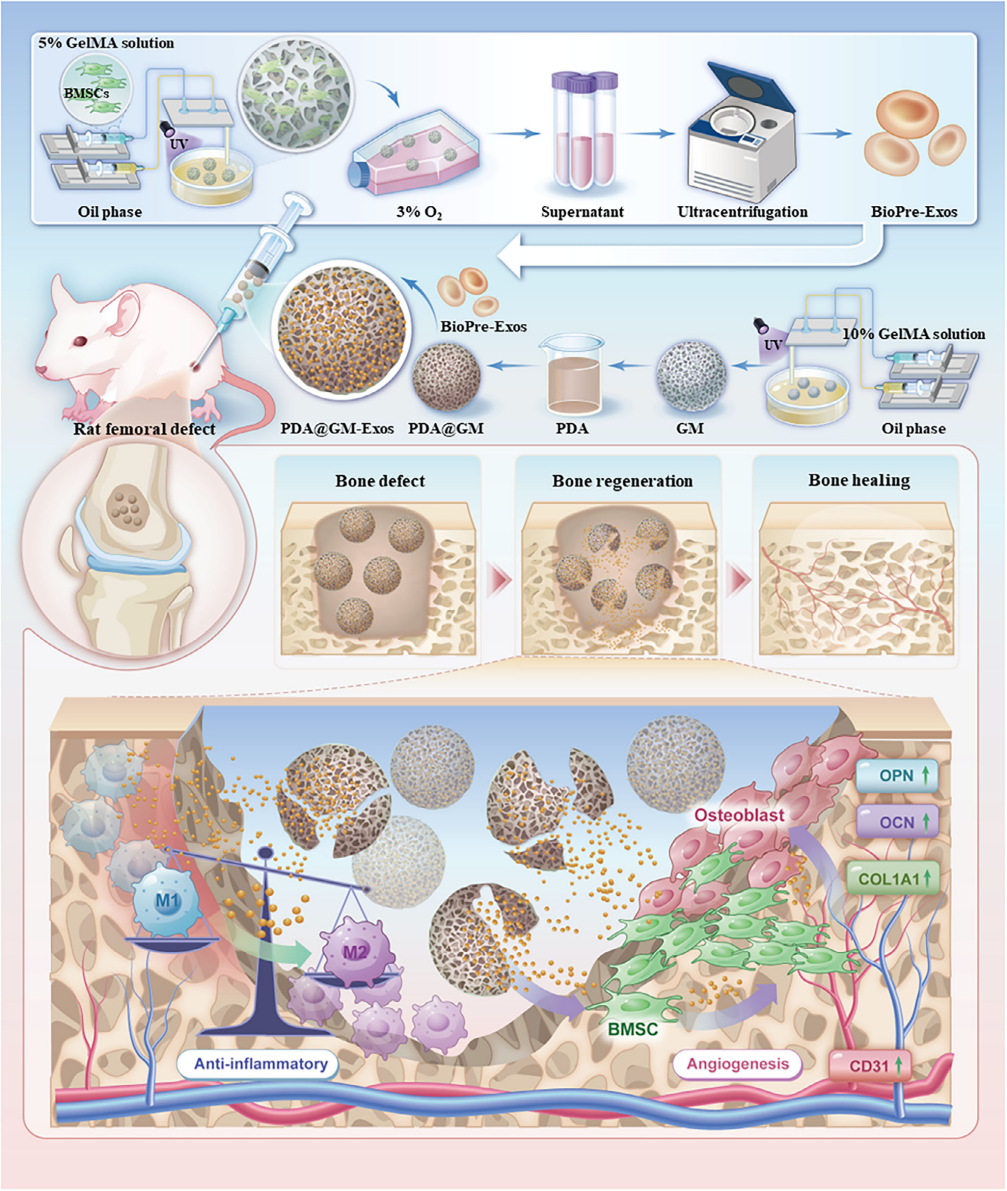
Schematic illustration of injectable PDA‐modified GelMA microspheres loaded with BioPre‐Exos for vascularized bone regeneration.

To address the aforementioned challenges, this study presents an innovative synergistic therapeutic system that combines biomimetic preconditioned BMSC‐derived exosomes (BioPre‐Exos) with injectable porous microspheres for the repair of critical‐sized bone defects (Figure 1). This system can effectively overcome the limitations of existing studies by integrating the dual advantages of injectable carriers and functionalized exosomes [8, 9, 19]. On the one hand, injectable porous microspheres not only enable minimally invasive delivery and conform to the irregular morphology of bone defects, but also provide a favorable microenvironment for endogenous cell infiltration and nutrient exchange at the defect site [20, 21]. After modification with polydopamine (PDA), they can further enhance exosome loading efficiency and achieve sustained controlled release, thereby avoiding rapid exosome clearance and resolving the burst release issue of traditional delivery systems [22, 23]. On the other hand, BioPre‐Exos were obtained by preconditioning BMSCs in a biomimetic bone marrow microenvironment constructed using 3% oxygen (O_2_) hypoxia combined with 3D gelatin methacryloyl (GelMA) microspheres, given that the physiological oxygen partial pressure of natural bone marrow ranges from 1% to 4% [24]. This culture system exhibits significant differences from traditional 2D normoxic (21% O_2_) [11, 12] or severely hypoxic (<1% O_2_) [10] conditions. Notably, 3D GelMA microspheres can mimic the collagenous extracellular matrix of native bone tissue, provide physiologically compatible adhesion and growth substrates for BMSCs, facilitate efficient nutrient exchange and metabolic waste elimination for encapsulated BMSCs [22, 23], and synergize with 3% O_2_ hypoxia to enhance the paracrine function of BMSCs [25]. Importantly, the obtained BioPre‐Exos could regulate key biological processes including BMSC migration, osteogenic differentiation, endothelial tube formation, and macrophage polarization toward an anti‐inflammatory phenotype, thereby providing synergistic regenerative signals for bone repair. Overall, this bifunctional system integrated the controlled release advantage of injectable microspheres and the bioactive signaling function of BioPre‐Exos, showing considerable potential for enhancing bone regeneration efficacy and facilitating clinical translation.

To fabricate this synergistic therapeutic system, we first prepared GelMA microspheres encapsulating rat BMSCs via microfluidic technology [26], which were then preconditioned in a 3% O_2_ environment for 72 h prior to culturing the cells for an additional 48 h in exosome‐depleted serum medium. The supernatant was then collected to extract BioPre‐Exos via ultracentrifugation [9]. Subsequently, injectable porous GelMA microspheres were fabricated via microfluidic technology [26], modified with PDA to enhance exosome loading efficiency and biocompatibility [22], and then loaded with BioPre‐Exos to construct the final therapeutic system. In vitro experiments demonstrate that this system markedly promoted BMSC migration and osteogenic differentiation, enhanced the tube formation capacity of human umbilical vein endothelial cells (HUVECs), and effectively induced the conversion of macrophages from a pro‐inflammatory to an anti‐inflammatory phenotype. In vivo studies confirm that it significantly accelerated bone formation and vascularization at the femoral defect site in rats. Collectively, the injectable microsphere/BioPre‐Exos system provides a novel and efficient therapeutic strategy for critical‐sized bone defects, offering potential implications for the clinical translation of orthopedic tissue engineering.

## 2 Results and Discussion

### 2.1 Preparation and Characterization of the Composite System

Critical‐sized bone defects remain a persistent clinical challenge due to their inadequate intrinsic regenerative capacity [3, 27, 28]. BMSC‐derived exosomes (BMSC‐Exos) have emerged as promising cell‐free therapeutics for osteoregeneration [8, 14]. However, their clinical translation is impeded by multiple core bottlenecks: short in vivo half‐life, rapid clearance, low delivery efficiency, poor targeting ability to defect sites, and insufficient osteogenic potency under single pretreatment regimens, which fail to support the long‐term repair of critical‐sized bone defects [11, 14, 15, 18]. To address these issues, we constructed a PDA‐coated GelMA microsphere (PDA@GM) carrier system for loading BioPre‐Exos, exosomes derived from BMSCs pretreated via 3D GelMA microsphere culture combined with 3% hypoxia. We then systematically characterized the components and comprehensive properties of this composite system.

**FIGURE 2:**
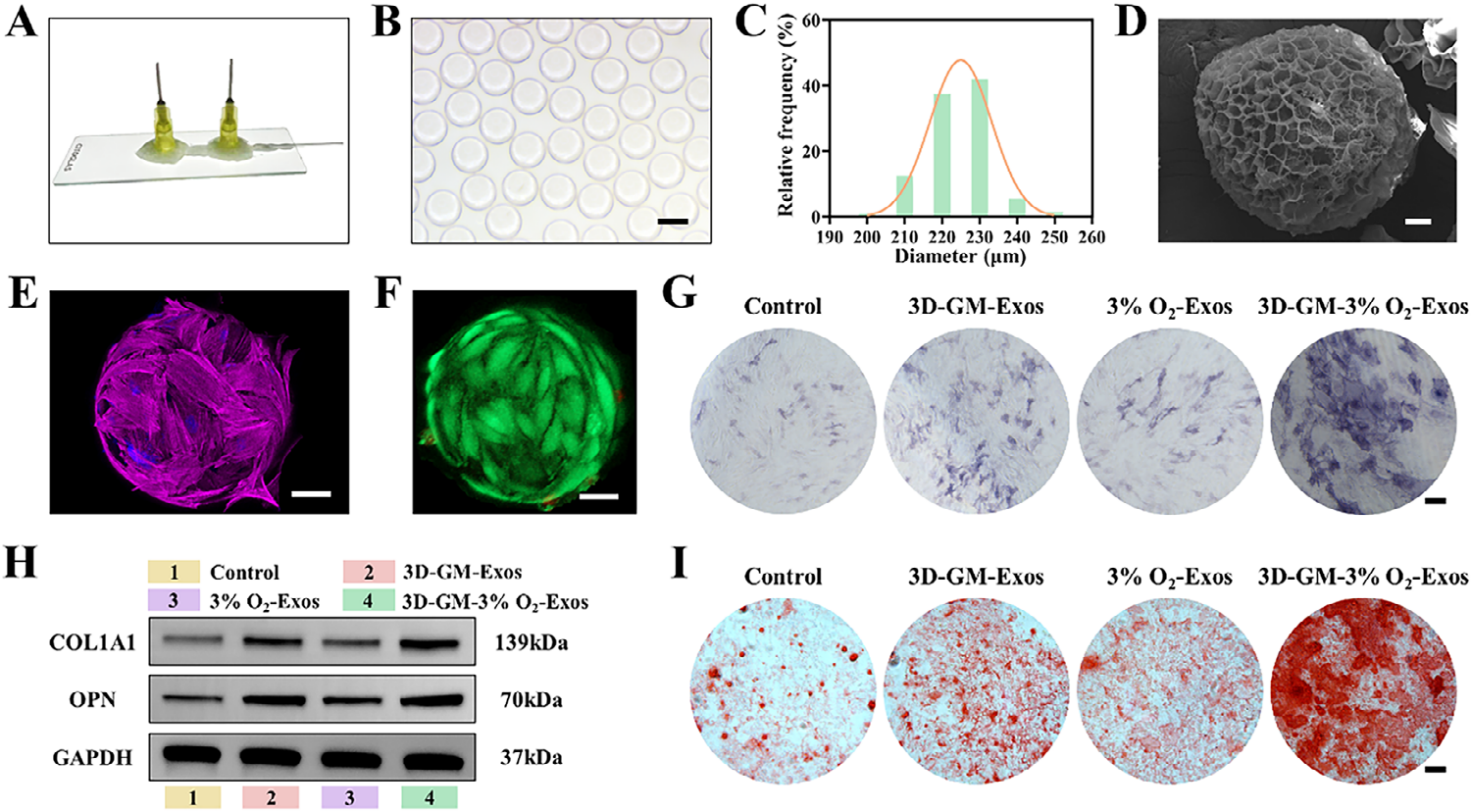
Fabrication, characterization, and cell assays of 5% GelMA microspheres. (A) Photograph of the microfluidic device for GelMA microsphere fabrication. (B) Optical micrograph of 5% GelMA microspheres. Scale bar: 200 µm. (C) Particle size distribution histogram of 5% GelMA microspheres. (D) SEM image of 5% GelMA microspheres. Scale bar: 25 µm. (E) Phalloidin staining of BMSCs cultured in 5% GelMA microspheres. Scale bar: 50 µm. (F) Live/dead staining of BMSCs cultured in 5% GelMA microspheres. Scale bar: 50 µm. (G) ALP staining of BMSCs treated with exosomes secreted by BMSCs under different pretreatment conditions at 1 week of osteogenic induction. Scale bar: 100 µm. (H) Western blotting analysis of COL1A1 and OPN in the above BMSCs at 2 weeks of osteogenic induction. (I) ARS staining of mineralized nodules in BMSCs treated with exosomes secreted by BMSCs under different pretreatment conditions at 3 weeks of osteogenic induction. Scale bar: 100 µm.

GelMA microspheres were fabricated using a microfluidic device (Figure 2A), a technique that precisely ensures uniform particle size distribution [26]. This uniformity represents a core advantage for 3D cell culture, as it enables homogeneous cell encapsulation efficiency, balanced nutrient supply, and synchronized cellular behaviors [29, 30]. It also effectively circumvents the drawbacks of traditional 2D culture (e.g., unidirectional cell polarity, impaired intercellular communication) [11] and heterogeneous microspheres (e.g., uneven cell distribution, poor experimental reproducibility) [22]. Optical micrographs of 5% GelMA microspheres (Figure 2B) exhibited regular spherical morphology, and the corresponding particle size distribution histogram (Figure 2C) revealed an average diameter of 223.6 ± 8.0 µm. Scanning electron microscopy (SEM) images (Figure 2D) further confirmed a porous architecture, which provides ample channels for cell adhesion and nutrient exchange [22]. It also facilitates efficient excretion of metabolic waste, thereby creating a biomimetic microenvironment for long‐term cell survival and function maintenance [23].

To verify the feasibility of GelMA microspheres for BMSC culture, BMSC‐laden 5% GelMA microspheres were prepared by mixing BMSCs with GelMA prepolymer solution via the aforementioned microfluidic method, followed by 3 days of incubation under 3% hypoxia. Phalloidin staining (Figure 2E) demonstrated that BMSCs spread well inside the microspheres, forming interconnected cellular networks. Live/dead staining (Figure 2F) confirmed high cell viability with a minimal dead cell ratio. These findings indicate that the 3D porous GelMA microenvironment possesses excellent biocompatibility and can stably support BMSC survival under hypoxic conditions, laying a solid foundation for generating BioPre‐Exos with enhanced osteogenic activity.

Exosomes secreted by BMSCs under different pretreatment conditions were isolated via differential ultracentrifugation [25]. Their osteogenic potential was evaluated using alkaline phosphatase (ALP) staining, Western blotting (WB), and alizarin red S (ARS) staining (Figure 2G–I). Compared with exosomes derived from BMSCs subjected to 3D microsphere culture or 3% hypoxia alone, BioPre‐Exos (i.e., the 3D‐GM‐3% O_2_‐Exos group) significantly enhanced ALP activity (Figure 2G), a key marker of early osteogenic differentiation [9]. This result was consistent with the upregulated expression of osteogenic‐related proteins collagen type I alpha 1 chain (COL1A1), and osteopontin (OPN) detected by WB (Figure 2H). ARS staining (Figure 2I) further confirmed a marked increase in calcium deposition in the BioPre‐Exos‐treated group. Collectively, these data demonstrate that the dual pretreatment strategy of 3D microsphere culture combined with 3% hypoxia synergistically enhances the osteogenic efficacy of BMSC‐Exos. This synergy is mainly attributed to the regulation of dual biomimetic microenvironments. On the one hand, the porous 3D GelMA microenvironment mimics the native stem cell niche (rich in collagen matrix), establishes physiological cell‐matrix interactions, and maintains BMSC phenotypic stability [22]. On the other hand, 3% O_2_ was selected as a physiologically relevant hypoxic preconditioning level, as it falls within the physiological oxygen range of native bone marrow (1%–4%) [24, 25] and such physiological hypoxic preconditioning has been reported in several studies to enhance both the osteogenic and angiogenic bioactivity of BMSC‑derived exosomes [10, 31, 32]. Together, they activate the hypoxia‐inducible factor (HIF) signaling pathway, drive the reprogramming of exosome secretion profiles toward a pro‐osteogenic and pro‐angiogenic phenotype, and strengthen osteoregeneration regulation [9, 25].

**FIGURE 3:**
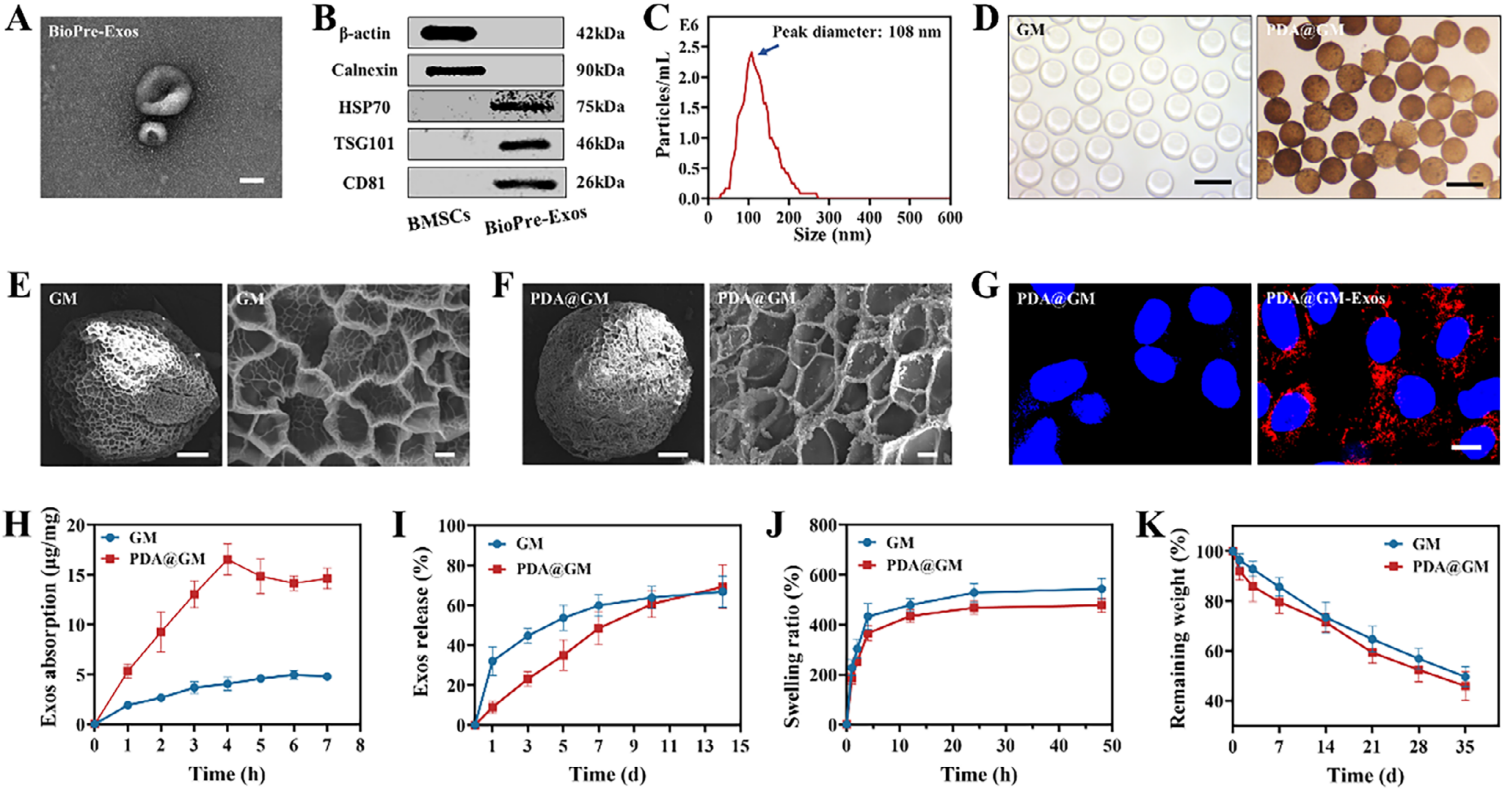
Identification of BioPre‐Exos and performance characterization of the PDA@GM composite system. (A) TEM image of BioPre‐Exos. Scale bar: 50 nm. (B) WB analysis of exosome markers (Hsp70, TSG101, and CD81) and negative controls (Calnexin, β‐actin). (C) NTA analysis for the size distribution of BioPre‐Exos. (D) Optical micrograph of GM and PDA@GM microspheres. Scale bar: 200 µm. (E, F) SEM images of GM and PDA@GM microspheres. Scale bars: 25 µm (left), 2 µm (right). (G) Confocal laser scanning microscopy image of DiD‐labeled BioPre‐Exos internalized by BMSCs. Scale bar: 25 µm. (H, I) Exosome adsorption capacity (n = 3) and sustained‐release profiles (n = 3) of GM and PDA@GM microspheres. (J, K) Swelling ratio (n = 3) and degradation curves (n = 3) of GM and PDA@GM microspheres in phosphate‐buffered saline (PBS). All data are presented as mean ± standard deviation (SD).

Subsequently, BioPre‐Exos were isolated and identified to ensure their authenticity and purity. Transmission electron microscopy (TEM) images (Figure 3A) showed that BioPre‐Exos exhibited a typical cup‐shaped morphology, a hallmark structural feature of exosomes [15]. WB analysis (Figure 3B) confirmed the expression of exosome‐specific markers TSG101, CD81, and Hsp70 in BioPre‐Exos [11]. The absence of calnexin (an endoplasmic reticulum marker) and β‐actin (a cytoskeletal marker) ruled out contamination by cellular debris or intact cells [9]. Nanoparticle tracking analysis (NTA) results (Figure 3C) indicated that BioPre‐Exos had an average particle size of 108 nm, which falls within the typical size range of exosomes (30–150 nm) [9], further verifying the successful isolation of high‐purity exosomes. We further characterized 10% GelMA microspheres (GM) and their PDA‐modified versions (PDA@GM). Optical micrographs (Figure 3D) showed that both maintained regular spherical shapes, with average diameters of approximately 141.3 ± 9.9 µm and 137.3 ± 12.3 µm, respectively (Figure S1). Meanwhile, SEM images (Figure 3E,F) revealed that both retained porous architectures, and PDA modification slightly increased the surface roughness of the microspheres. PDA modification exerted a negligible effect on particle size, thereby well preserving the injectable property. Figure S2 confirmed that the microspheres could be injected into a “Z” shape, highlighting their potential for minimally invasive therapy of irregular bone defects [21]. The porous structure increases exosome binding sites via physical adsorption [22]. Protonated amino groups on PDA carry a weak positive charge, forming electrostatic attraction with negatively charged exosomes, while catechol groups simultaneously form hydrogen bonds [23]. These three mechanisms synergistically enhance binding capacity, significantly improving the exosome adsorption efficiency of PDA@GM. This was further verified by confocal laser scanning microscopy images (Figure S3), which showed successful loading of 1,1'‐dioctadecyl‐3,3,3',3'‐tetramethylindodicarbocyanine (DiD)‐fluorescently labeled BioPre‐Exos onto PDA@GM surfaces.

Confocal laser scanning microscopy observations (Figure 3G) revealed strong DiD fluorescence signals inside BMSCs co‐cultured with PDA@GM‐BioPre‐Exos, indicating efficient exosome internalization by cells. We further evaluated the exosome adsorption and sustained‐release properties of GM and PDA@GM (Figure 3H,I). The results showed that PDA@GM had a higher adsorption capacity and exhibited a more desirable sustained‐release profile, with continuous exosome release over 14 days. In contrast, GM showed a “burst release followed by plateau” pattern. Swelling and degradation assays (Figure 3J,K) demonstrated that PDA@GM had a moderate swelling ratio, enabling adaptation to the in vivo microenvironment without causing excessive local tissue pressure [23]. It degraded slightly faster than GM, and this degradation rate is highly matched with the timeline of bone regeneration [23]. This ensures carrier structural stability during continuous exosome delivery while being gradually resorbed and replaced by newly formed bone tissue, thus avoiding long‐term foreign body reactions [22]. Taken together, the PDA@GM‐BioPre‐Exos composite system, with its synergistically enhanced adsorption‐sustained release performance, excellent biocompatibility, and reinforced osteogenic activity, provides a highly promising cell‐free therapeutic strategy for clinical repair of critical‐sized bone defects.

### 2.2 Biocompatibility Evaluation of PDA@GM

**FIGURE 4:**
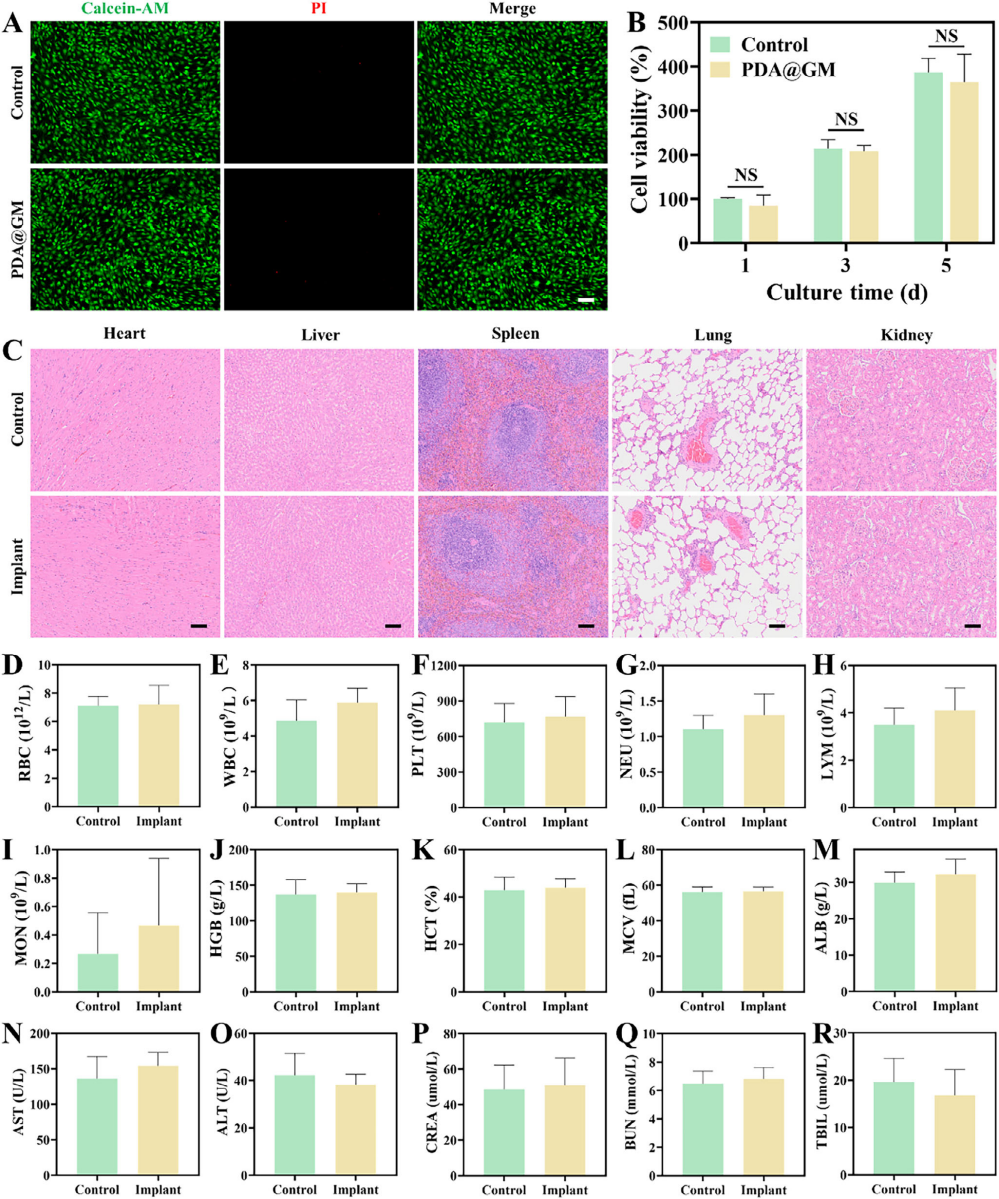
Biocompatibility evaluation of PDA@GM microspheres in vitro and in vivo. (A) Calcein‐AM/PI staining of BMSCs treated with PDA@GM extracts for 5 days. Scale bar: 100 µm. (B) CCK‐8 assay of BMSCs cultured with PDA@GM extracts (Control vs PDA@GM, n = 3). A two‐tailed unpaired Student's t‐test was used for statistical analysis, and no significant difference was observed. (C) Histological sections of major organs after 3‐week subcutaneous implantation of PDA@GM in rats. Scale bar: 100 µm. (D‐L) Hematological analysis indices of rats (Control vs Implant, n = 3). No statistical analysis was performed as all values were within normal physiological ranges. (M‐R) Hepatic/renal function indices of rats (Control vs Implant, n = 3). No statistical analysis was performed as all values were within normal physiological ranges. All data are presented as mean ± SD. NS (not significant) for *p* > 0.05.

Biocompatibility serves as a fundamental prerequisite for the clinical translation of biomaterials, and is particularly critical for bone tissue engineering applications involving long‐term in vivo implantation [9]. This study comprehensively assessed the cellular and systemic biocompatibility of PDA@GM microspheres through in vitro cell experiments and in vivo subcutaneous implantation models. Results of calcein acetoxymethyl ester/propidium iodide (Calcein‐AM/PI) staining (Figure 4A) showed that the number of viable BMSCs exposed to PDA@GM microsphere extracts for 5 days was comparable to that in the control group, with only a negligible number of dead cells observed in both groups. Results of the Cell Counting Kit‐8 (CCK‐8) assay (Figure 4B) confirmed that the cell proliferation rate of the PDA@GM group was consistent with the untreated control group, verifying that PDA@GM exhibits no cytotoxicity [6].

To evaluate the long‐term in vivo biocompatibility of PDA@GM microspheres, the microspheres were subcutaneously implanted into the dorsal region of rats for 3 weeks, with a control group (no material implantation) established for comparison (Figure 4C). Histological analysis of major organs revealed that all tissues in the PDA@GM implantation group maintained normal histological structures, with no abnormalities such as inflammation, necrosis, or fibrosis detected. Additionally, hematological analysis was performed to evaluate the impact of PDA@GM implantation on the host's blood system (Figure 4D–L), and hepatic/renal function indices were tested to assess its metabolic toxicity (Figure 4M–R), and neither assay showed significant abnormalities. Collectively, the in vitro and in vivo experimental results consistently confirm that PDA@GM microspheres possess excellent biocompatibility, characterized by an absence of cytotoxicity, proliferation inhibition, and systemic tissue damage or metabolic disorders in the host.

### 2.3 Multifunctional Effects of the PDA@GM‐BioPre‐Exos Composite System

**FIGURE 5:**
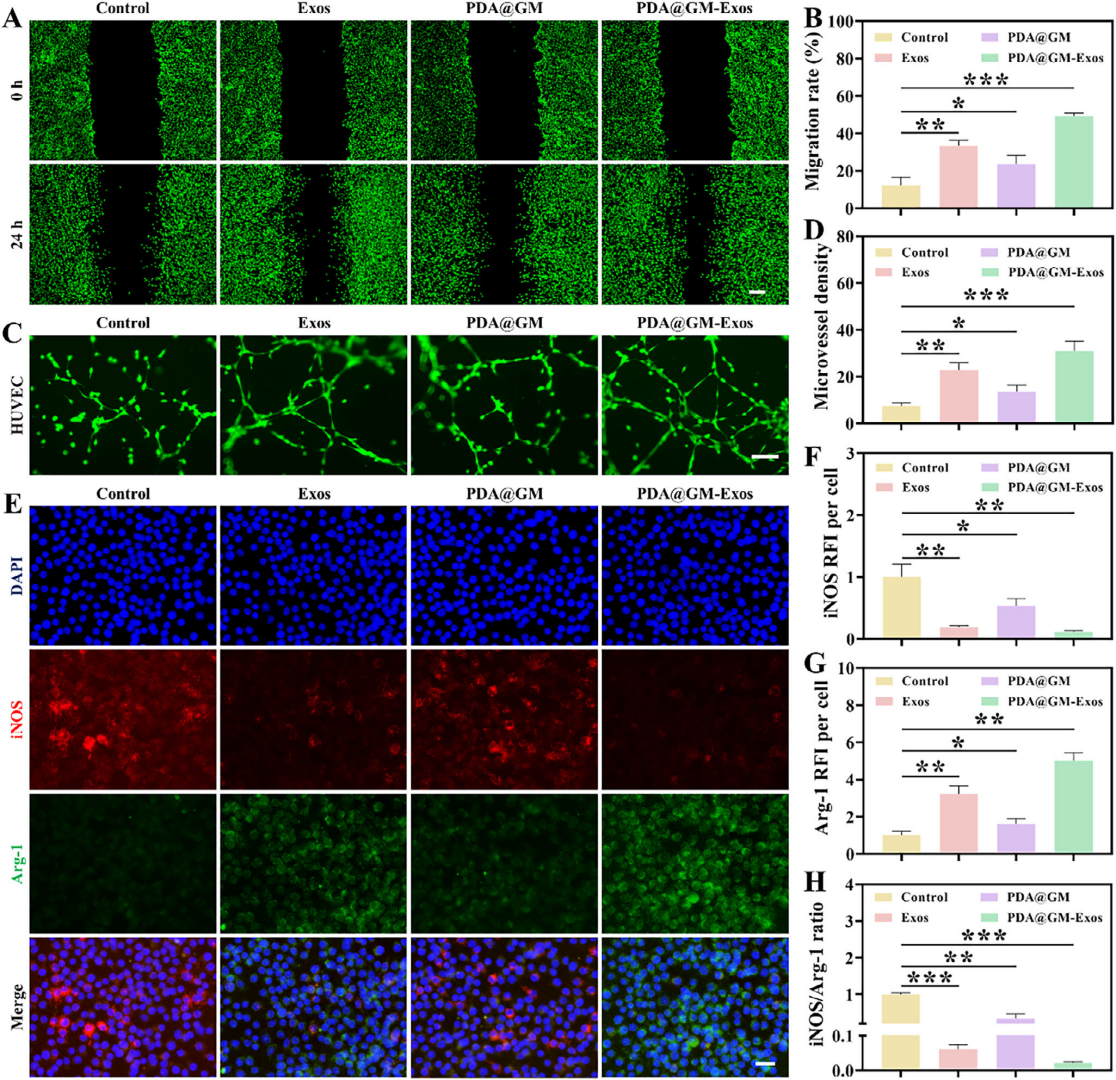
Multifunctional effects of the PDA@GM‐BioPre‐Exos composite system in vitro. (A) Representative images of the scratch wound healing assay at 0 and 24 h for evaluating the effect of the composite system on BMSC migration. Scale bar: 200 µm. (B) Quantitative analysis of migration rate derived from the scratch wound healing assay (n = 3). (C, D) Representative images of HUVEC tube formation assay and quantitative analysis of microvessel density to assess the angiogenic capacity of the composite system (n = 3). Scale bar: 100 µm. (E–H) Immunofluorescence staining of iNOS and Arg‐1 in RAW264.7 macrophages, along with quantitative analysis of RFI and the iNOS/Arg‐1 ratio to examine macrophage polarization regulated by the composite system (n = 3). Scale bar: 25 µm. All data are presented as mean ± SD. Statistical significance was determined using one‐way analysis of variance (ANOVA) with Tukey's post hoc test. ***p < 0.001, **p < 0.01, *p < 0.05.

The ability to modulate cellular behaviors (e.g., stem cell migration, endothelial angiogenesis, and macrophage polarization) is crucial for establishing a favorable osteoinductive microenvironment, which serves as a vital foundation for bone tissue repair [33]. This study evaluated the multifunctional properties of the PDA@GM‐BioPre‐Exos composite system through a series of in vitro experiments. A scratch wound healing assay (Figure 5A) was performed to assess the system's effect on BMSC migration, with quantitative analysis of migration rate (Figure 5B) validating the experimental conclusions. Results showed that the wound closure rate of the PDA@GM‐Exos group reached 49.2 ± 1.6%, significantly higher than that of the Control group (12.2 ± 4.3%), Exos group (33.4 ± 2.9%), and PDA@GM group (23.5 ± 4.7%) (*p* < 0.05), while the latter two groups exhibited only a moderate migration‐promoting effect. This synergistic modulation stems from the coordinated interplay of the composite system, where the PDA coating of PDA@GM within the system modulates cell adhesion signaling pathways to create a suitable microenvironment for exosome‐mediated BMSC migration, and exosomes in turn carry bioactive molecules to facilitate BMSC migration with the two components working in tandem to amplify the migration‐promoting efficacy [22, 23].

A HUVEC tube formation assay was employed to evaluate the angiogenic capacity of the composite system, with quantitative microvessel density (MVD) analysis further corroborating the results (Figure 5C,D). The MVD of the PDA@GM‐Exos group was 30.9 ± 4.1 cells/mm^2^, significantly higher than that of the other three groups (Control group: 7.2 ± 1.5 cells/mm^2^, Exos group: 22.7 ± 3.1 cells/mm^2^, PDA@GM group: 13.6 ± 2.7 cells/mm^2^, *p* < 0.05), and this group formed more intact and dense tubular networks. The porous structure of PDA@GM in the composite system enables efficient loading and sustained release of exosomes, ensuring the full exertion of exosomal bioactivity, and exosomes can deliver pro‐angiogenic factors to activate downstream signaling pathways [23]. These two components synergistically enhance angiogenic efficiency, providing critical support for in vitro vascular formation.

Immunofluorescence staining of inducible nitric oxide synthase (iNOS) and arginase‐1 (Arg‐1) in RAW264.7 macrophages combined with quantitative analysis of relative fluorescence intensity (RFI) and the iNOS/Arg‐1 ratio (Figure 5E–H) was conducted to explore the composite system's regulatory effect on macrophage polarization. Results demonstrated that the PDA@GM‐Exos group exhibited the lowest iNOS RFI (0.11 ± 0.02 fold change) and the highest Arg‐1 RFI (5.01 ± 0.43 fold change) with significant differences compared to the Control group (*p* < 0.01). The corresponding iNOS/Arg‐1 ratio of the PDA@GM‐Exos group reached 0.02 ± 0.00, indicating a significant shift toward M2 polarization. The Control group presented a distinct M1 polarization phenotype. The Exos group induced a moderate M2 polarization effect with an iNOS/Arg‐1 ratio of 0.06 ± 0.01 fold change (*p* < 0.001) while the PDA@GM group slightly promoted M2 polarization with a ratio of 0.34 ± 0.12 fold change (*p* < 0.01). This regulatory effect arises from the synergism of the composite system. The PDA coating of PDA@GM exerts inherent anti‐inflammatory properties to inhibit excessive activation of M1 macrophages, and the loaded exosomes further induce M2 polarization by secreting anti‐inflammatory cytokines [22, 23]. This dual synergistic action collectively modulates the immune microenvironment and eliminates inflammatory barriers to facilitate bone regeneration. Collectively, the PDA@GM‐BioPre‐Exos composite system offers a promising functional strategy for bone tissue repair by synergistically regulating stem cell migration, angiogenesis, and macrophage polarization.

### 2.4 Osteogenic Promotion Efficacy of the PDA@GM‐BioPre‐Exos Composite System In Vitro

**FIGURE 6:**
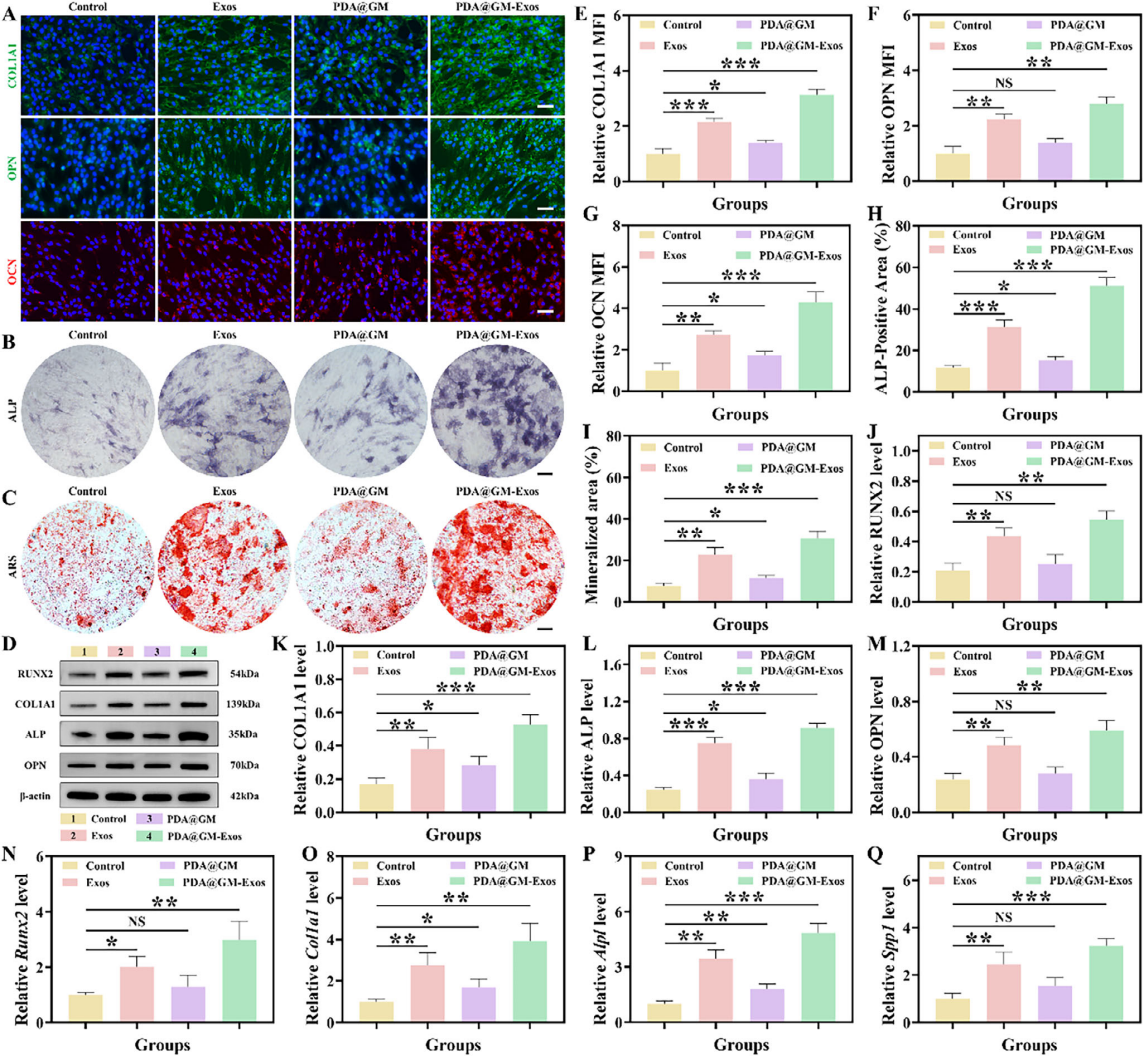
In vitro osteogenic differentiation of BMSCs regulated by the PDA@GM‐BioPre‐Exos composite system. (A) Immunofluorescence staining for osteogenic markers in BMSCs at distinct osteogenic induction stages: COL1A1 (early stage) at 1 week, OPN (mid‐stage) at 2 weeks, and OCN (late stage) at 3 weeks post‐induction. (B) ALP staining for BMSCs at the early osteogenic stage. (C) ARS staining for evaluating mineralized nodule formation in BMSCs at 3 weeks post‐induction. (D) WB analysis of core osteogenic regulatory factors and markers, including RUNX2, COL1A1, ALP, and OPN. (E–G) Quantitative analysis of relative MFI for COL1A1 (n = 3), OPN (n = 3) and OCN (n = 3) corresponding to (A). (H) Quantitative analysis of ALP‐positive area percentage (n = 3) corresponding to (B). (I) Quantitative analysis of mineralized area percentage (n = 3) corresponding to (C). (J–M) Quantitative analysis of relative expression levels of RUNX2 (n = 3), COL1A1 (n = 3), ALP (n = 3) and OPN (n = 3) proteins corresponding to (D). (N–Q) qPCR analysis of mRNA expression levels of osteogenesis‐related genes, including *Runx2* (n = 3), *Col1a1* (n = 3), *Alpl* (n = 3) and *Spp1* (n = 3). All data are presented as mean ± SD. Statistical significance was determined using ANOVA with Tukey's post hoc test. ***p < 0.001, **p < 0.01, *p < 0.05, NS for *p* > 0.05.

To clarify the osteogenic potential of the PDA@GM‐BioPre‐Exos composite system, a series of in vitro osteogenic induction assays were carried out in BMSCs to determine the expression differences of core osteogenic markers at distinct stages of osteogenic differentiation (Figure 6A–Q). Figure 6A shows the immunofluorescence staining results of BMSCs at different osteogenic induction stages, where the early‐stage marker COL1A1 was detected at 1 week, the mid‐stage marker OPN at 2 weeks, and the late‐stage marker osteocalcin (OCN) at 3 weeks post‐induction [34]. Quantitative analysis of the relative mean fluorescence intensity (MFI) of these markers is presented in Figure 6E–G. The relative. MFI of COL1A1, OPN, and OCN in the PDA@GM‐Exos group reached 3.1 ± 0.2, 2.7 ± 0.2, and 4.2 ± 0.5 fold compared with the control group, respectively, which were markedly elevated relative to the control, Exos, and PDA@GM groups. COL1A1 serves as the structural basis for bone matrix deposition, OPN mediates cell‐matrix interactions during osteogenesis, and OCN is closely linked to bone mineralization [9]. The significant upregulation of these three markers indicates that the composite system can accelerate the osteogenic differentiation of BMSCs. This outcome arises from the synergistic crosstalk of components within the system, as the PDA coating establishes a biocompatible microenvironment for cell adhesion and differentiation, while the loaded exosomes deliver osteogenic bioactive molecules to target and upregulate the expression of osteogenic markers [22, 23]. Consistently, results of early ALP staining (Figure 6B,H) demonstrated that the percentage of ALP‐positive area in the PDA@GM‐Exos group was the highest among all groups, reaching 51.0 ± 3.9%, which was significantly higher than that in the control group (11.8 ± 0.8%), Exos group (31.4 ± 3.3%) and PDA@GM group (15.2 ± 1.8%). Complementarily, quantitative colorimetric assay of relative ALP activity further confirmed this trend, with the PDA@GM‐Exos group exhibiting the highest relative ALP activity (Figure S4). As a classic early osteogenic marker, ALP activity directly reflects the initiation of osteogenic differentiation [5]. The notable increase in ALP‐positive area in this group is mutually confirmed by the concurrent COL1A1 immunofluorescence results, further validating the time‐dependent osteogenic promotion effect of the system.

To assess the late‐stage osteogenic capacity of the composite system, ARS staining was performed at 3 weeks post‐induction to evaluate the bone mineralization ability of BMSCs (Figure 6C,I). The PDA@GM‐Exos group formed more and denser mineralized nodules, with the corresponding mineralized area percentage reaching 30.7 ± 3.3%, which was significantly higher than that in the control group (7.7 ± 1.4%), Exos group (22.7 ± 1.4%) and PDA@GM group (11.5 ± 1.2%). Bone mineralization represents the terminal stage of osteogenic differentiation, and the remarkably enhanced mineralization capacity of this group indicates that the composite system can not only initiate the osteogenic differentiation of BMSCs but also further promote osteoblast maturation and mineralized matrix formation [5, 9]. This superior late‐stage osteogenic efficacy is associated with the efficient loading and sustained release properties of the PDA@GM carrier for exosomes, which maintains a stable and continuous osteogenic signaling microenvironment for cells throughout the entire induction period.

At the molecular level, WB analysis was conducted to detect the protein expression levels of core osteogenic regulatory factors and markers, including runt‐related transcription factor 2 (RUNX2), COL1A1, ALP, and OPN (Figure 6D,J–M). The relative expression levels of these target proteins in the PDA@GM‐Exos group were the highest among all groups, with the expression levels of RUNX2, COL1A1, ALP, and OPN reaching 2.6 ± 0.2, 3.0 ± 0.3, 3.7 ± 0.2 and 2.4 ± 0.3 relative to the control group, respectively, and being significantly higher than those in the control, Exos, and PDA@GM groups. RUNX2 acts as a master transcription factor regulating the expression of downstream osteogenic markers, and its significant upregulation suggests that the composite system modulates the osteogenic differentiation of BMSCs at the transcriptional level [12]. The coordinated upregulation of RUNX2, COL1A1, ALP, and OPN at the protein level further confirms that the osteogenic promotion efficacy of the system is not targeted at a single marker but a systematic and synergistic regulation of the entire osteogenic signaling pathway. Additionally, quantitative real‐time polymerase chain reaction (qPCR, Figure 6N–Q) was used to detect the mRNA expression levels of osteogenesis‐related genes, including runt‐related transcription factor 2 (*Runx2*), collagen type I alpha 1 chain (*Col1a1*), alkaline phosphatase (*Alpl*), and secreted phosphoprotein 1 (*Spp1*). The mRNA expression levels of these genes in the PDA@GM‐Exos group were significantly upregulated compared with the control group, with *Runx2*, *Col1a1*, *Alpl*, and *Spp1* reaching 2.9 ± 0.6, 3.9 ± 0.8, 4.8 ± 0.5, and 3.2 ± 0.3 fold, respectively. The expression trends of these genes were highly consistent with the corresponding protein levels, indicating that the PDA@GM‐BioPre‐Exos composite system mediates the osteogenic differentiation of BMSCs through a multi‐level regulatory mode from transcriptional activation to protein synthesis. This regulatory pattern ensures the stability and effectiveness of the osteogenic effect, which constitutes the core advantage of the composite system over the single‐component Exos or PDA@GM systems.

In conclusion, in vitro osteogenic experiments confirm that the PDA@GM‐BioPre‐Exos composite system can significantly promote the osteogenic differentiation of BMSCs. Its mechanism of action relies on the synergistic effect between the PDA coating and exosomes within the system, as they coordinately upregulate the expression of osteogenic markers at both the gene and protein levels, effectively initiate the early stage of osteogenic differentiation, and markedly enhance late‐stage mineralization capacity. Through this comprehensive regulation, the system optimizes the osteogenic microenvironment and activates the osteogenic signaling pathway, laying a solid experimental foundation for subsequent in vivo bone defect repair studies.

### 2.5 PDA@GM‐BioPre‐Exos Composite System Promote Femur Defect Repair in Vivo

**FIGURE 7:**
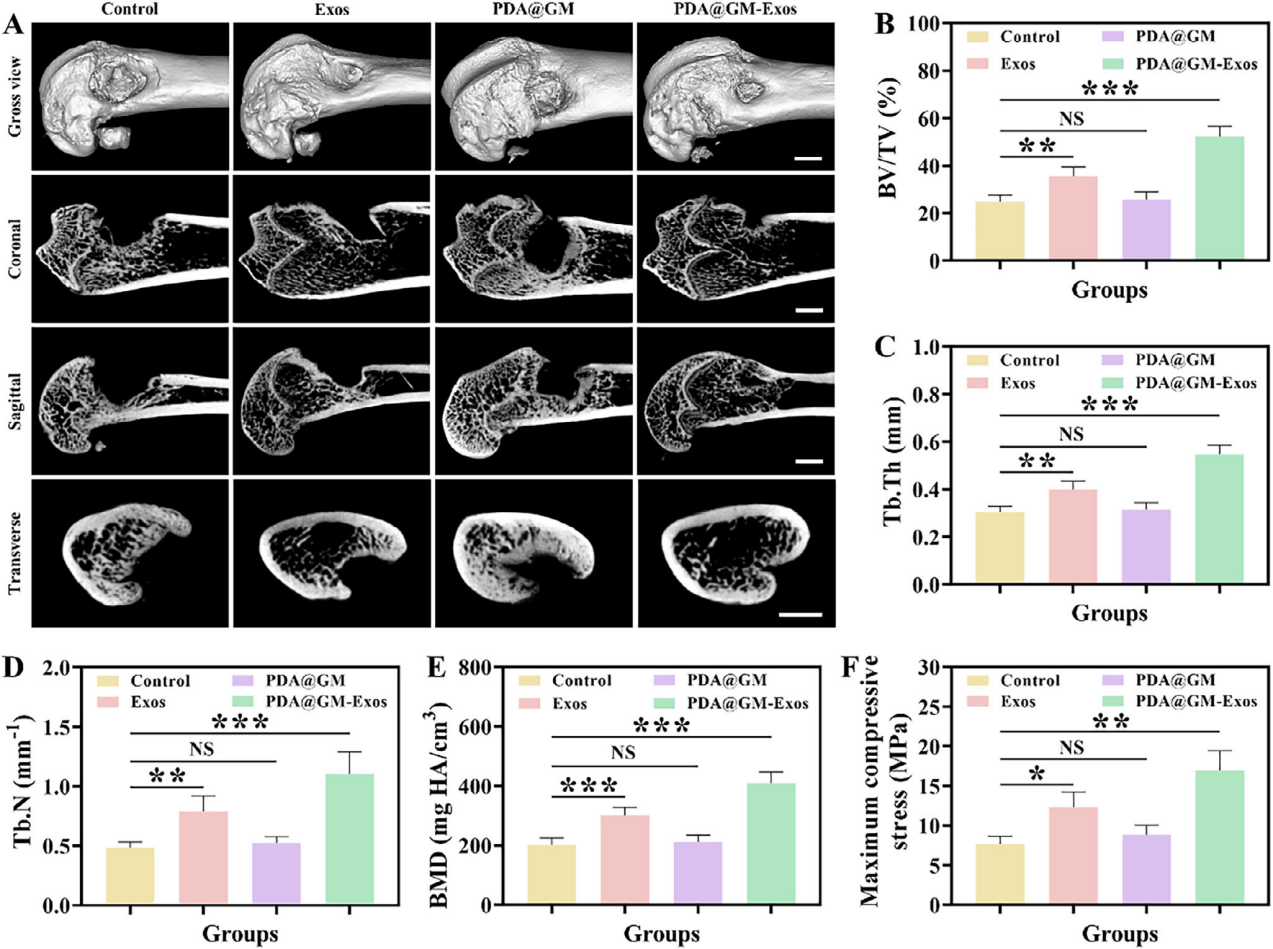
In vivo evaluation of bone defect repair using micro‐CT and biomechanical testing. (A) Representative 3D reconstructed micro‐CT images and coronal, sagittal, and transverse sectional views of femoral defects in each group at 12 weeks postoperatively. All scale bars are 2 mm. (B‐E) Quantitative analysis of bone structural parameters, including BV/TV (n = 5), Tb.Th (n = 5), Tb.N (n = 5), and BMD (n = 5). (F) Maximum compressive stress of the defect region determined by biomechanical testing (n = 3). All data are presented as mean ± SD. Statistical significance was determined using ANOVA with Tukey's post hoc test. ***p < 0.001, **p < 0.01, *p < 0.05, NS for *p* > 0.05.

Building on the in vitro osteogenic potential of the PDA@GM‐BioPre‐Exos composite system, we established a rat critical‐sized femoral metaphyseal defect model [5, 35]. This model was used to systematically validate the composite system's in vivo bone defect repair efficacy and to provide experimental evidence for subsequent preclinical translation. It was selected for its widespread use and established recognition in vascularized bone regeneration research [5, 36, 37, 38], as its cancellous bone characteristics facilitate the evaluation of bone regeneration capacity and mirror clinical scenarios of isolated femoral metaphyseal defects [5, 38]. While we acknowledge that the femoral metaphyseal defect model has richer vascularity than mid‐diaphyseal cortical bone defects [5, 39], it allows us to validate our claims about the composite system's angiogenic properties in a clinically relevant setting [38]. This is critical for bridging preclinical research and clinical application. We recognize that a less vascularized model (e.g., a mid‐diaphyseal critical‐sized defect) would provide a more rigorous test [39]. Thus, we plan to address this in future studies by using mid‐diaphyseal femoral defects to further verify the composite system's angiogenic potential under more challenging hypovascular conditions. At 12 weeks post‐surgery, micro‐computed tomography (micro‐CT), biomechanical testing, histological staining, and immunofluorescence staining were performed to systematically evaluate new bone formation, bone structural quality, mechanical properties, and the osteogenesis‐angiogenesis coupling regulatory effect in the defect region. Figure 7A presented the 3D reconstructed images and coronal, sagittal, transverse sectional images of the femoral defect region at 12 weeks post‐surgery. The PDA@GM‐Exos group exhibited the most prominent bone defect repair efficacy, with the defect region almost completely filled by newly formed bone tissue with dense and continuous trabeculae that formed an obscure and tight connection with the host bone. In contrast, a large unhealed gap with scarce new bone formation was observed in the control group, while the Exos and PDA@GM groups only showed partial defect filling with limited new bone tissue and sparse, discontinuous bone trabeculae. This repair phenotype directly reflected the synergistic osteogenic advantage of the composite system. Specifically, PDA@GM injectable microspheres provide a basic physical support via space‐filling to facilitate cell adhesion and osteogenic differentiation, and the sustained release of exosomes continuously delivers osteogenic bioactive signals to regulate the functional activity of endogenous osteogenesis‐related cells [15, 22]. Quantitative analysis of key bone structural parameters in the defect region (Figure 7B–E) showed that the bone volume/total volume (BV/TV), trabecular thickness (Tb.Th), trabecular number (Tb.N), and bone mineral density (BMD) in the PDA@GM‐Exos group were 52.3 ± 4.2%, 0.54 ± 0.03 mm, 1.10 ± 0.18 mm^−^^1^ and 410.3 ± 37.1 mg HA/cm^3^, respectively, which were significantly higher than those in the Exos group (35.5 ± 3.9%, 0.39 ± 0.03 mm, 0.78 ± 0.13 mm^−^^1^, 301.3 ± 26.4 mg HA/cm^3^), PDA@GM group (25.7 ± 3.3%, 0.31 ± 0.02 mm, 0.52 ± 0.05 mm^−^^1^, 211.0 ± 23.7 mg HA/cm^3^) and control group (24.8 ± 2.7%, 0.30 ± 0.02 mm, 0.48 ± 0.04 mm^−^^1^, 201.2 ± 23.8 mg HA/cm^3^). BV/TV and BMD are direct indicators of new bone formation and mineralization degree, and their significant increase indicates that the composite system can markedly improve the efficiency of new bone formation and extracellular matrix mineralization [9, 33, 40], which was consistent with the enhanced mineralization effect observed in in vitro ARS staining. The elevation of Tb.Th and Tb.N confirms that the newly formed bone has a more stable structural integrity, which is attributed to the systematic regulation of the composite system on the entire process of osteogenic differentiation [5]. Biomechanical testing results (Figure 7F) showed that the maximum compressive stress in the defect region of the PDA@GM‐Exos group was 16.9 ± 2.5 MPa, which was significantly higher than that in the Exos group (12.2 ± 1.9 MPa), PDA@GM group (8.8 ± 1.2 MPa) and control group (7.6 ± 0.9 MPa). Thus, the newly formed bone in the PDA@GM‐Exos group possessed excellent mechanical bearing capacity and could effectively restore the mechanical function of the defective femur [9, 41]. This result was closely associated with the high‐quality bone trabecular structure and sufficient mineralization in this group [42]. PDA@GM microspheres fill the defect gap and form a physical support to prevent defect collapse, thereby creating a stable microenvironment for early osteogenesis [22]. Subsequently, the mineralized new bone gradually strengthens the stability of the defect site, forming a positive cycle of “microsphere space‐filling support – osteogenic repair – mechanical function recovery” [7, 14].

**FIGURE 8:**
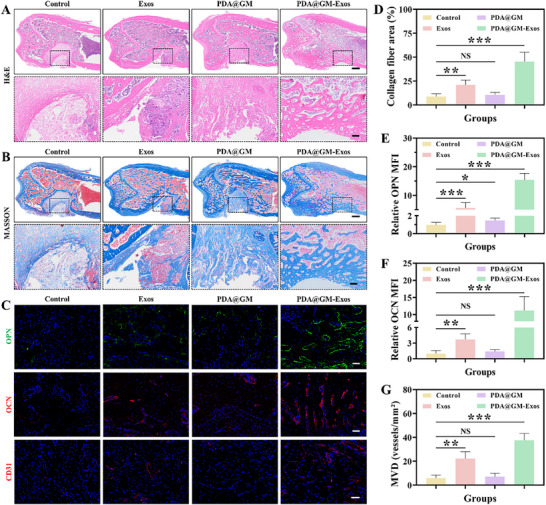
Histological and immunofluorescence analysis of bone defect repair. (A) H&E staining of femoral defect sections in each group at 12 weeks postoperatively. Representative images are shown at two magnifications with scale bars of 1 mm (upper panel) and 200 µm (lower panel). (B) Masson's trichrome staining of femoral defect sections in each group at 12 weeks postoperatively. Representative images are shown at two magnifications with scale bars of 1 mm (upper panel) and 200 µm (lower panel). (C) Representative immunofluorescence images showing the expression of OPN (osteogenesis), OCN (mineralization), and CD31 (angiogenesis) in the defect area, with all images captured at 1.5 mm from the defect edge. All scale bars are 50 µm. (D) Quantitative analysis of the percentage of newly formed collagen fiber area from Masson's trichrome staining (n = 5). (E‐G) Quantitative analysis of the relative MFI of OPN (n = 5) and OCN (n = 5), and the MVD of CD31‐positive blood vessels (n = 5). All data are presented as mean ± SD. Statistical significance was determined using ANOVA with Tukey's post hoc test. ***p < 0.001, **p < 0.01, *p < 0.05, NS for *p* > 0.05.

Histological staining results (Figure 8A,B,D) showed that under hematoxylin‐eosin (H&E) staining, a large amount of new bone tissue was observed in the defect region of the PDA@GM‐Exos group with only a small amount of residual fibrous connective tissue, while the control group was dominated by loose fibrous tissue with rare new bone formation, and the Exos and PDA@GM groups had limited new bone tissue with sparse bone matrix deposition. Under Masson's trichrome staining, the collagen fibers in the PDA@GM‐Exos group were more concentrated and significantly higher in content, whereas the other three groups had low collagen fiber content and limited regenerative capacity. Quantitative analysis of the area ratio of newly formed collagen fibers revealed that the ratio in the PDA@GM‐Exos group was 45.3 ± 9.8%, which was significantly higher than that in the Exos group (21.0 ± 4.9%), PDA@GM group (10.5 ± 2.7%) and control group (8.8 ± 3.0%). Collagen fibers are the core structural component of bone matrix, and the increase in their deposition provides a solid structural foundation for bone regeneration, which further verifies the bone repair efficacy of the composite system at the histological microlevel [8, 18, 39].

Immunofluorescence staining results (Figure 8C,E–G) showed that the fluorescence signal intensities of OPN and osteocalcin OCN in the defect region of the PDA@GM‐Exos group were significantly higher than those in the other three groups. Quantitative analysis demonstrated that the relative MFI of OPN and OCN in the PDA@GM‐Exos group were 15.3 ± 2.3 and 11.1 ± 4.1 fold those in the control group, respectively (all *p* < 0.001), which confirms that the composite system can sustainably promote osteoblast maturation and bone mineralization [3, 6, 36]. In terms of angiogenesis, the PDA@GM‐Exos group exhibited the highest MVD in the defect region, which was significantly higher than that in the control group (*p* < 0.001). Its CD31‐positive blood vessels were densely distributed around the newly formed bone to supply sufficient nutrients and oxygen for bone regeneration, thereby realizing the coupling regulation of osteogenesis and angiogenesis [43, 44, 45]. This feature constitutes the core advantage of the composite system over single‐component materials in repairing bone defects [46].

In conclusion, the rat femoral defect model experiments confirmed that the PDA@GM‐BioPre‐Exos composite system can significantly promote the repair of rat femoral bone defects, and its core mechanism originates from the synergistic effect between PDA@GM microspheres and exosomes. PDA@GM microspheres fill the defect gap and form a physical support to maintain the stability of the local microenvironment, thus providing a structural foundation for osteogenic repair. Meanwhile, the sustainedly released exosomes can regulate the functional activity of endogenous osteogenesis‐related cells and simultaneously promote angiogenesis in the defect region. The synergistic effect of the two components achieves the elevation of new bone formation, optimization of bone structure, and recovery of bone mechanical function. This study provides solid experimental evidence for the preclinical research and application of the PDA@GM‐BioPre‐Exos composite system as a novel biomaterial for bone defect repair.

## 3 Conclusion

In summary, this study innovatively constructed a novel bioactive composite system, PDA@GM‐BioPre‐Exos, which significantly enhances bone defect repair via the strategic integration of injectable PDA@GM microspheres and therapeutic exosomes. The PDA@GM microspheres serve a dual function. They act as a supportive scaffold for cellular attachment and tissue ingrowth, and as an efficient delivery platform facilitating the sustained release of exosomes, thereby overcoming the limitations of rapid clearance of free exosomes in vivo. As the primary bioactive component, BioPre‐Exos play a pivotal role in promoting the osteogenic differentiation of endogenous progenitor cells, enhancing angiogenesis, and modulating macrophage behavior within the defect microenvironment. When evaluated in a rat femoral defect model, the PDA@GM‐BioPre‐Exos system exhibited remarkable efficacy in promoting new bone formation and the development of a functional vascular network. These findings highlight the considerable potential of this composite system as an innovative therapeutic strategy for clinical bone defect repair and provide a fresh perspective for the design of advanced biomaterials in regenerative medicine.

Despite the promising outcomes, this research has certain inherent limitations that warrant further exploration. First, the efficacy of the PDA@GM‐BioPre‐Exos system was only evaluated in a rodent model, and its translational potential requires further validation in large animal models that more closely mimic human bone physiology. Second, the specific exosomal components mediating the observed osteogenic, angiogenic, and immunomodulatory effects, as well as their detailed molecular mechanisms, remain to be fully elucidated. Future studies will therefore focus on conducting preclinical investigations in sheep or pig models to assess long‐term biosafety and bone regeneration efficacy in long‐segment diaphyseal cortical bone defects, and on deciphering the precise molecular mechanisms to further optimize the composite system, thus paving the way for its eventual clinical translation.

## 4 Materials and Methods

### 4.1 Materials

All chemical reagents, histological staining kits, molecular biology reagents, cell culture components, and assay kits were obtained from commercial suppliers unless otherwise stated, with GelMA synthesized in‐house. Specifically, methylacrylic anhydride and gelatin for GelMA synthesis were purchased from Sigma–Aldrich (Germany), while 2‐hydroxy‐2‐methylpropiophenone (HMPP) and dopamine were from Aladdin Industrial Corporation (China), Span 80 from Sigma–Aldrich, and liquid paraffin from Macklin (China). ARS solution was obtained from Procell Life Science & Technology Co., Ltd. (China), whereas H&E and Masson's trichrome staining kits were supplied by Servicebio (China). For cell culture experiments, Alpha Minimum Essential Medium (α‐MEM), Dulbecco's Modified Eagle's Medium (DMEM, Gibco, USA), exosome‐depleted fetal bovine serum (FBS, Vivacell, China), and basement membrane matrix (MedChemExpress, China) were utilized. Corresponding cellular assay kits, including Calcein‐AM/PI, CCK‐8, DiD membrane staining kit, bicinchoninic acid (BCA) protein assay kit, and ALP colorimetric activity assay kit, were purchased from Beyotime Biotechnology, along with SYBR Green qPCR Mix, and phalloidin as well as TRIzol reagent were obtained from Invitrogen (Thermo Fisher Scientific, USA). Antibodies used included those targeting β‐actin, Calnexin, HSP70, TSG101, CD81 (Abcam, UK), glyceraldehyde‐3‐phosphate dehydrogenase (GAPDH, Servicebio), the osteogenic markers RUNX2, COL1A1, OPN, OCN, ALP, and CD31 (Servicebio), and the macrophage polarization markers iNOS and Arg‐1 (eBioscience, Thermo Fisher Scientific). Immunoblotting supplies, including polyvinylidene fluoride (PVDF) membranes and enhanced chemiluminescence (ECL) substrate, were provided by Servicebio to complete experimental workflows.

### 4.2 Synthesis of GelMA

GelMA was synthesized as follows. Briefly, 5 g of gelatin was dissolved in 50 mL of PBS at 50°C under continuous stirring until fully dissolved. Then, 3 mL of methacrylic anhydride was added, and the reaction was maintained at 50°C for 3 h with constant stirring. The mixture was centrifuged at 8000 rpm for 10 min to remove unreacted residues, and the supernatant was collected and diluted with two volumes of preheated ultrapure water to quench the reaction. The product was dialyzed against ultrapure water at 37°C for 7 days using a dialysis membrane with a molecular weight cutoff of 14 kDa, and then lyophilized to obtain white foamy GelMA powder, which was stored at −20°C in the dark.

### 4.3 Fabrication of GelMA Microspheres via Microfluidic Device

5% and 10% (w/v) GelMA microspheres were fabricated using a custom‐fabricated microfluidic droplet device. The oil phase consisted of liquid paraffin supplemented with 5% (v/v) Span 80. The dispersed phase was a pre‐prepared GelMA aqueous solution, dissolved in PBS with 0.5% HMPP as the photoinitiator, with only the GelMA concentration adjusted for the two groups. For 5% GelMA microspheres, the aqueous phase flow rate was set to 10 µL/min and the oil phase to 100 µL/min to yield uniform microspheres with an average diameter of ∼223 µm. For 10% GelMA microspheres, the aqueous phase flow rate was adjusted to 6 µL/min with the oil phase maintained at 100 µL/min to produce uniform microspheres with an average diameter of ∼141 µm. After emulsification, the microsphere droplets were photo‐crosslinked under ultraviolet light for 30 s to solidify the GelMA network, followed by repeated centrifugation and PBS washing to separate them from the oil phase and remove residual oil and surfactant. Subsequently, the overall morphology of the purified microspheres was observed using a bright‐field microscope (Nikon Corporation, Japan), and the diameter distribution of the microspheres was measured.

### 4.4 Scanning Electron Microscope

Microstructural characterization was conducted via a SEM (Hitachi TM‐1000, Japan) operated at an accelerating voltage of 12 kV for 5% GM, 10% GM, and PDA@GM subjected to gradient cooling followed by freeze‐drying. Prior to imaging, all microsphere samples were sputter‐coated with gold to enhance surface conductivity and improve imaging clarity.

### 4.5 Culture and Staining of BMSC‐Laden GelMA Microspheres

Rat BMSCs were incorporated into 5% (w/v) GelMA precursor solution (0.5% HMPP in PBS) at 1×10^6^ cells/mL, then fabricated into ∼223 µm microspheres as described above. The cell‐laden microspheres were cultured in a 3% hypoxic environment for 3 days to facilitate BMSC spreading, adhesion, and initial proliferation. Afterward, F‐actin staining was performed by fixing cells with 4% paraformaldehyde for 15 min and permeabilizing with 0.2% Triton X‐100 in PBS for 5 min. They were then incubated with Alexa Fluor 488‐conjugated phalloidin for 20 min, and nuclei were counterstained with 4',6‐diamidino‐2‐phenylindole (DAPI) before fluorescent images were captured using a Zeiss LSM 900 confocal microscope. Live‐dead staining was subsequently conducted with Calcein‐AM/PI. The staining solution was incubated at 37°C for 30 min, and images were acquired using the same confocal microscope to evaluate cellular viability in 3D microspheres.

### 4.6 Isolation and Characterization of BMSC‐Derived Exosomes

For BMSCs subjected to biomimetic pretreatment (3% hypoxic culture in 5% GelMA microspheres; derived exosomes referred to as BioPre‐Exos), after 3‐day incubation, the cells were maintained in exosome‐depleted medium supplemented with 10% exosome‐free FBS for 48 h to enhance exosome secretion. Subsequently, the conditioned medium was processed for exosome isolation via sequential centrifugation steps: 2,000 × g (10 min) to remove floating cells, 10,000 × g (30 min) for debris clearance, 0.22 µm membrane filtration, and final ultracentrifugation at 100,000 × g (70 min) to pellet exosomes. Afterward, the purified exosomes were resuspended in PBS for comprehensive characterization. Size distribution and concentration were quantified by NTA (ZetaView PMX‐110, Particle Metrix, Germany). Meanwhile, morphology was observed via TEM (JEOL JEM‐1400Plus, Japan) at 100 kV with 2% uranyl acetate negative staining. Additionally, WB was performed to confirm exosomal identity using markers TSG101, CD81, and Hsp70 (1:1000 dilution), with β‐actin (1:5000) and Calnexin (1:1000) as controls to exclude cytoplasmic and endoplasmic reticulum contamination, respectively.

### 4.7 Osteogenic Assays of BMSC‐Derived Exosomes under Various Pretreatments

To evaluate the osteogenic induction capacity of BMSC‐derived exosomes under different pretreatment conditions, four experimental groups were established: a Control group (without exosome treatment), a 3D‐GM‐Exos group (exosomes from BMSCs cultured in 3D GelMA microspheres under normoxia), a 3% O_2_‐Exos group (exosomes from BMSCs with 3% hypoxia pretreatment alone), and a 3D‐GM‐3% O_2_‐Exos group (exosomes from BMSCs with combined 3D GelMA microsphere culture and 3% hypoxia pretreatment, namely BioPre‐Exos). The osteogenic potential of exosomes in each group was assessed via three approaches, with detailed protocols provided in subsequent sections, including ALP staining at 1 week post‐induction, ARS staining at 3 weeks post‐induction, and WB at 2 weeks post‐induction. For WB assays, COL1A1 (1:1000 dilution) and OPN (1:1000 dilution) were designated as target osteogenic proteins, while GAPDH was employed as the internal reference.

### 4.8 Exosome Adsorption and Release Assays

For surface modification, the 10% GM prepared above were incubated in a dopamine solution (1 mg/mL, dissolved in 10 mM Tris‐HCl buffer at pH 8.5) for 12 h to obtain PDA@GM. To visualize exosome loading onto the microspheres, BioPre‐Exos were fluorescently tagged with DiD using a commercial cell membrane labeling kit. Briefly, exosome pellets were reconstituted in the kit's membrane staining working solution and incubated at 37°C for 15 min. The staining reaction was terminated by adding exosome‐depleted FBS. The labeled exosomes were then pelleted by centrifugation, loaded onto PDA@GM microspheres, and visualized via confocal laser scanning microscopy (Zeiss LSM900, Germany). To compare the exosome adsorption capacities of pristine GM and PDA@GM, 5 mL of BioPre‐Exos with a concentration of 1000 µg/mL was mixed with 100 mg of either GM or PDA@GM, followed by incubation at 4°C for 1–7 h. To investigate the exosome adsorption kinetics (n = 3), 100 µL of the supernatant was collected and concentrated at preset time points (1, 2, 3, 4, 5, 6, and 7 h post‐adsorption). For the establishment of exosome release profiles of the microsphere‐exosome complexes (n = 3), 10 mg of the GM/BioPre‐Exos or PDA@GM/BioPre‐Exos composites were immersed in 100 µL of PBS and incubated on a horizontal shaker at 37°C with a rotation speed of 60 rpm for 14 days. At predetermined time intervals (days 1, 3, 5, 7, 10, and 14 post‐incubation), 80 µL of the supernatant was harvested, and an equal volume of fresh PBS was replenished into the system immediately. The exosome concentration in the collected supernatant was determined using a BCA protein quantification kit.

### 4.9 Uptake of Exosomes by BMSCs

BMSCs were subjected to co‐culture with PDA@GM loaded with DiD‐labeled BioPre‐Exos in complete medium for 6 h. To evaluate the internalization of exosomes by BMSCs, laser scanning confocal microscopy (Zeiss LSM900) was employed.

### 4.10 Swelling Ratio Assay

Freeze‐dried GM and PDA@GM (10 mg each, n = 3) were soaked in PBS at 37°C. At preset time points (1, 2, 4, 12, 24, 48 h post‐soaking), the microspheres were retrieved, gently blotted to remove surface PBS, and weighed. The swelling ratio was calculated using the wet and initial dry weights.

### 4.11 Microsphere Degradation Assay

Freeze‐dried GM and PDA@GM microspheres (100 mg per sample, n = 3) were subjected to shaken incubation in PBS at 37°C. At predetermined time points (1, 3, 7, 14, 21, 28, and 35 days post‐incubation), the microspheres were retrieved, lyophilized, and weighed to obtain the dry weight. The residual weight percentage was subsequently calculated.

### 4.12 Cytotoxicity and Proliferation Assays

To evaluate the cytocompatibility of PDA@GM, BMSCs were cultured in α‐MEM containing PDA@GM extracts for 5 days, followed by live/dead staining with Calcein‐AM/PI (incubation at 37°C for 30 min) and observation under a Leica DMi8 fluorescence microscope (Germany). For proliferation kinetics quantification, BMSCs were cultured in the same extract‐supplemented α‐MEM for 1, 3, and 5 days (n = 3), then incubated with CCK‐8 solution at 37°C for 2 h. Absorbance at 450 nm was subsequently detected using a microplate spectrophotometer (Thermo Scientific Multiskan FC, USA).

### 4.13 Biocompatibility of PDA@GM in Vivo

Adult Sprague‐Dawley rats (n = 3) received subcutaneous implantation of PDA@GM at the dorsal region. Three weeks post‐implantation, the rats were euthanized, and whole blood, serum samples, as well as visceral organs were retrieved. Whole blood analysis was carried out using an automatic hematology analyzer (Mindray BC‐2800vet, China), and liver and kidney functions were evaluated from serum via an automatic biochemical analyzer (Rayto Life Technology, China). Visceral tissue specimens were fixed in 4% paraformaldehyde, processed through paraffin embedding and sectioning, and then subjected to H&E staining. Digital images of the stained sections were acquired using a digital slide scanner (Olympus VS200, Japan).

### 4.14 Scratch Assay

BMSC migration was assessed via scratch assays. Cells were randomly divided into four groups (n = 3 per group): Control (untreated cells), Exos (cells treated with BioPre‐Exos), PDA@GM (cells co‐cultured with PDA@GM), and PDA@GM‐Exos (cells co‐cultured with PDA@GM‐Exos). Uniform scratches were created with a sterile pipette tip. Calcein‐AM staining was performed on cells at 0 and 24 h post‐scratch, and images were acquired via a Leica DMi8 microscope. Cell migration rates were calculated as the scratch closure percentage.

### 4.15 Endothelial Tubulogenesis Assay

HUVECs were reconstituted in DMEM and assigned to the same four groups as the scratch assay. Cells (5×10^4^ cells/well) were seeded into 24‐well plates pre‐coated with basement membrane matrix and cultured for 12 h. At the endpoint, HUVECs were stained with Calcein‐AM at 37°C for 30 min, followed by fluorescent image acquisition via a Leica DMi8 fluorescence microscope to assess tubular network formation. Vascular tube density was quantified as the number of lumens per square millimeter (n = 3).

### 4.16 Immunofluorescence Staining for Macrophage Polarization

RAW264.7 macrophages were plated on glass coverslips at 1×10^5^ cells/well, stimulated to M1 polarization with 100 ng/mL lipopolysaccharide (LPS) for 12 h, and rinsed with PBS. They were divided into the aforementioned four groups (n = 3 per group) and incubated at 37°C with 5% CO_2_ for 72 h. For co‐immunofluorescence staining, cells were fixed in 4% paraformaldehyde for 15 min, blocked with immunofluorescence blocking solution for 1 h, and incubated with primary antibodies anti‐iNOS (1:200) and anti‐Arg‐1 (1:100) at 4°C overnight. Nuclei were counterstained with DAPI for 5 min. Fluorescent images were captured via a confocal microscope, and RFI of iNOS and Arg‐1 per cell was quantified with ImageJ software to assess M1‐M2 polarization.

### 4.17 Osteogenic Differentiation Assay

BMSCs were resuspended in α‐MEM and randomly distributed into the same four experimental groups as aforementioned (n = 3 per group). Osteogenic differentiation was induced with osteogenic induction medium for the set time periods.

### 4.18 Quantitative Real‐Time PCR

After 7 days of osteogenic induction, total RNA was extracted from BMSCs with TRIzol reagent. Complementary DNA (cDNA) was synthesized by reverse transcription using the Evo M‐MLV RT Mix Kit, and qPCR was performed with SYBR Green Master Mix. Primer sequences are provided in Table S1. mRNA expression levels were normalized against GAPDH via the comparative 2^−ΔΔCt^ method.

### 4.19 Immunofluorescence Staining of BMSCs

BMSCs (n = 3) were plated on glass coverslips, subjected to osteogenic differentiation, and harvested at 1, 2, and 3 weeks for COL1A1, OPN, and OCN staining, respectively. Cells were fixed in 4% paraformaldehyde, blocked in immunostaining blocking solution, and incubated at 4°C overnight with primary antibodies against COL1A1, OPN, or OCN (1:500 dilution each). Following incubation with fluorochrome‐conjugated secondary antibodies, nuclei were counterstained with DAPI. Fluorescent images were captured using a fluorescence microscope, and MFI of each marker was quantitated with ImageJ software.

### 4.20 Western Blotting

After 14 days of induction, cells were disrupted with Radioimmunoprecipitation Assay (RIPA) buffer. Proteins were resolved via Sodium Dodecyl Sulfate–Polyacrylamide Gel Electrophoresis (SDS‐PAGE), transblotted onto PVDF membranes, and blocked in 5% non‐fat dry milk. Membranes were incubated overnight at 4°C with primary antibodies against RUNX2, COL1A1, OPN, ALP (1:1000 dilution), GAPDH (1:5000), and β‐actin (1:5000). They were then incubated with corresponding Horseradish peroxidase (HRP)‐conjugated secondary antibodies for 1 h. Protein bands were visualized using an ECL detection system and quantitated with AIWBwell software.

### 4.21 ALP Activity Assay

After 7 days of osteogenic induction, BMSCs (n = 3) were fixed in 4% paraformaldehyde for 20 min and incubated with ALP staining working solution for 30 min following the manufacturer's instructions. The ALP‐positive area ratio was quantified using ImageJ software. For quantitative analysis of ALP enzymatic activity, a colorimetric assay was performed using p‐nitrophenyl phosphate (pNPP) as the substrate, and the absorbance at 405 nm was measured with a microplate reader to determine relative ALP activity.

### 4.22 Mineralization Assay

On day 21 of osteogenic induction, calcium deposition was evaluated by ARS staining. Cells (n = 3) were fixed in 4% paraformaldehyde for 20 min, then stained with 2% ARS solution for 30 min, and observed under a Leica DMi8 inverted microscope. Mineralized nodules were quantified using ImageJ software.

### 4.23 Rat Femur Defect Model Preparation

All in vivo experimental procedures were reviewed and approved by the Institutional Animal Care and Use Committee of Zhejiang Center of Laboratory Animals (ZJCLA‐IACUC‐20011062). Twelve‐week‐old male Sprague Dawley rats (n = 32, 8 per group) were allowed a 2‐week preoperative acclimation period. Under isoflurane anesthesia, a 1‐cm longitudinal incision was made along the lateral left knee, and the distal femur was exposed via blunt dissection of thigh muscles. A 4 mm × 4 mm (diameter × depth) circular bone defect was then created in the lateral metaphysis using a hollow trephine drill. Defects were implanted with PDA@GM (with or without BioPre‐Exos incorporation). Control rats received no implantation, whereas the exosome‐only group received direct BioPre‐Exos injection into the defect. All animals received routine postoperative analgesia and antibiotic prophylaxis.

### 4.24 Micro‐Computed Tomography (Micro‐CT) Scan

At 12 weeks post‐surgery, twenty rats (n = 5 per group) were euthanized for micro‐CT assessment. Left femora were dissected and fixed in 4% paraformaldehyde for 48 h. Specimens were imaged with a high‐resolution micro‐CT scanner (NMC‐200, Pingseng Scientific, China) using standardized parameters: 80 kV tube voltage, 60 µA current, and 35 µm isotropic voxel resolution. 3D reconstructions were generated via the manufacturer's dedicated software. Key bone morphometric parameters including BV/TV, Tb.Th, Tb.N, and BMD were quantified using the Avatar Analysis Suite.

### 4.25 Mechanical Testing of Newly Formed Bone

At 12 weeks post‐surgery, 12 rats (n = 3 per group) were euthanized to evaluate the mechanical properties of newly formed bone within the femoral defect. Bone specimens encompassing the defect region were dissected, and test samples were processed by sectioning 1 mm proximal to the defect's superior margin and 1 mm distal to its inferior margin (preserving the intact medial condyle) using a low‐speed diamond saw. Uniaxial compression tests were performed on a materials testing system (MTS Standard 43) at a constant loading rate of 1 mm/min.

### 4.26 Histological and Immunofluorescence Assays

The femoral specimens that had undergone micro‐CT scanning from 20 rats were further processed for subsequent histological and immunofluorescence assays. All tissue specimens were uniformly decalcified in 0.5 M ethylenediaminetetraacetic acid (EDTA) solution followed by paraffin embedding. H&E and Masson's trichrome staining were carried out per standardized protocols, with histomorphometric images captured using a digital slide scanner. For immunofluorescence staining targeting OPN (1:500 dilution), OCN (1:1000 dilution), and CD31 (1:500 dilution), antigen retrieval was performed in Tris‐EDTA buffer (pH 8.0) at 95°C for 25 min. Tissue sections were blocked with 3% bovine serum albumin (BSA) for 30 min, incubated overnight at 4°C with the respective primary antibodies, and then incubated with fluorophore‐conjugated secondary antibodies for 1 h at room temperature, prior to DAPI counterstaining. All immunofluorescence images (n = 5) were acquired using a confocal laser scanning microscope.

### 4.27 Statistical Analysis

Data are presented as the mean ± SD. Prior to statistical testing, all data were evaluated for outliers (Grubbs’ test), normality (Shapiro‐Wilk test), and homogeneity of variance to validate the assumptions of parametric tests. No significant outliers or assumption violations were identified. For in vitro experiments, the sample size was n = 3 biological replicates per group. For in vivo animal experiments, the sample size was n = 5 biological replicates per group. Comparisons between two groups were performed using two‐tailed unpaired Student's t‐test. For multi‐group comparisons (four groups), ANOVA was applied, followed by Tukey's post hoc test. The significance level (α value) was set at 0.05 for all statistical tests. All statistical analyses were conducted using GraphPad Prism 8.0.2 (La Jolla, CA, USA). Statistical significance was defined as ***p < 0.001, **p < 0.01, *p < 0.05.

## Author Contributions

L. L., H.Z., L.S., and Y.S. contributed to writing – original draft, data curation, and conceptualization. L. L., H.Z., L.S., Y.S., Y.X., J.H., J.W. contributed to visualization, and methodology. L.Z., J.Z., J.M., and W.X. contributed to writing, review, editing, supervision, resources, and funding acquisition.

## Conflicts of Interest

The authors declare no conflicts of interest.

## Supporting information




**Supporting File**: advs74987‐sup‐0001‐SuppMat.docx.

## Data Availability

The data that support the findings of this study are available from the corresponding author upon reasonable request.
