# Supplementary material for: Injectable Porous Microspheres Loaded With Biomimetic Preconditioned Bone Marrow Mesenchymal Stem Cell‐Derived Exosomes for Vascularized Bone Regeneration
Source: Adv Sci (Weinh). 2026 Mar 24;13(32):e74987. doi: 10.1002/advs.74987 (PMC13252651; doi:10.1002/advs.74987)
Supplement: Supplementary file 1 — Supporting File: advs74987‐sup‐0001‐SuppMat.docx. [file ADVS-13-e74987-s001.docx]

**Supporting Information**

**Injectable Porous Microspheres Loaded with Biomimetic Preconditioned Bone Marrow Mesenchymal Stem Cell-Derived Exosomes for Vascularized Bone Regeneration**

*Lijun Li, Hao Zhang, Lingtong Sun, Yingfeng Su, Yang Xu, Jian Huang, Jingchao Wen, Jinjin Zhu*, Jianjun Ma*, and Wenbin Xu**

L. Li, Y. Su, J. Wen, J. Zhu, W. Xu

Department of Orthopaedic Surgery, Sir Run Run Shaw Hospital, Zhejiang University School of Medicine & Zhejiang Key Laboratory of Mechanism Research and Precision Repair of Orthopaedic Trauma and Aging Diseases, Hangzhou 310016, Zhejiang, China

Email: zjjspine003@zju.edu.cn; xuwenbin@zju.edu.cn

J. Ma

Department of Orthopaedic Surgery，the Fourth Affiliated Hospital of School of Medicine, and International School of Medicine, International Institutes of Medicine，Zhejiang University，Yiwu，China

Email: sealteam@zju.edu.cn

H. Zhang

College of Pharmaceutical Sciences, Zhejiang Chinese Medical University，Hangzhou 310053, China

L. Sun

Hangzhou Xixi Hospital Affiliated to Zhejiang Chinese Medical University, Hangzhou, 310023, Zhejiang, China

Y. Xu

Department of Thoracic Surgery, Zhejiang Cancer Hospital, Hangzhou, Zhejiang 310022, China

J. Huang

Department of Ultrasound, Sir Run Run Shaw Hospital, Zhejiang University School of Medicine

Lijun Li, Hao Zhang, Lingtong Sun, and Yingfeng Su contributed equally to this work.

**Table S1. The sequence of the RT-PCR primers.**

| Genes | Forward primer | Reverse primer |
| --- | --- | --- |
| *Runx2* | TACCCAGGCGTATTTCAGATGAT | TGTAAGTGAAGGTGGCTGGATAGT |
| *Col1a1* | CCCAGCGGTGGTTATGACTT | TCGATCCAGTACTCTCCGCT |
| *Spp1* | GAACAGTATCCCGATGCCACA | GTGTGTTTCCACGCTTGGTTC |
| *Alpl* | CGGCACCTGCCTTACCAACT | ACTGTGGAGACGCCCATACC |
| *Gapdh* | CTGGAGAAACCTGCCAAGTATG | GGTGGAAGAATGGGAGTTGCT |


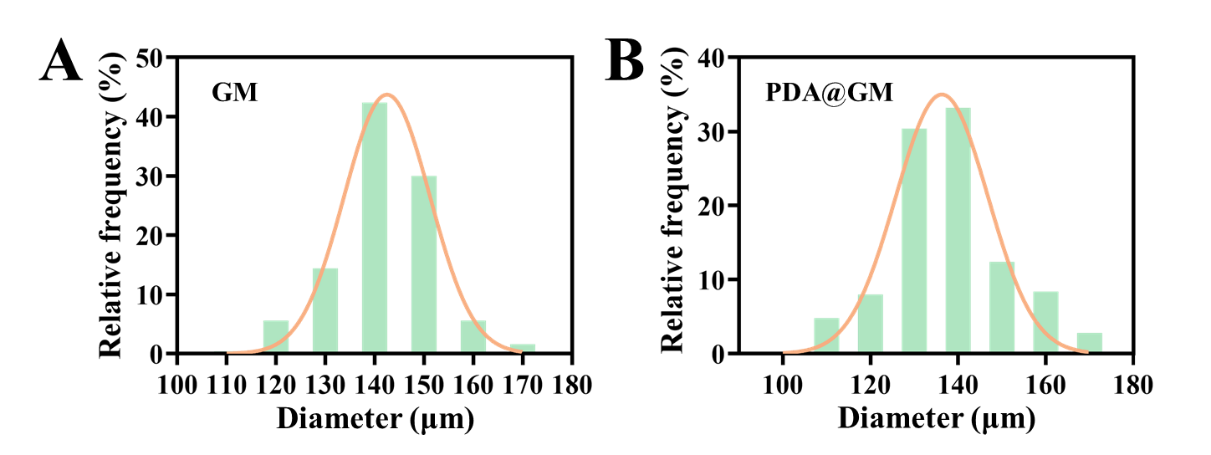


**FIGURE S1** Size distribution of GM and PDA@GM microspheres. (A) Particle size distribution histogram of GM. (B) Particle size distribution histogram of PDA@GM.


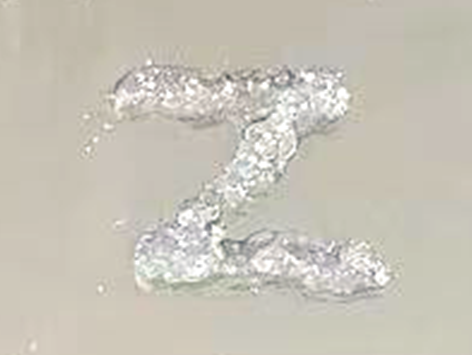


**FIGURE S2** Injectable GM microspheres fabricated into a Z-shape upon injection.


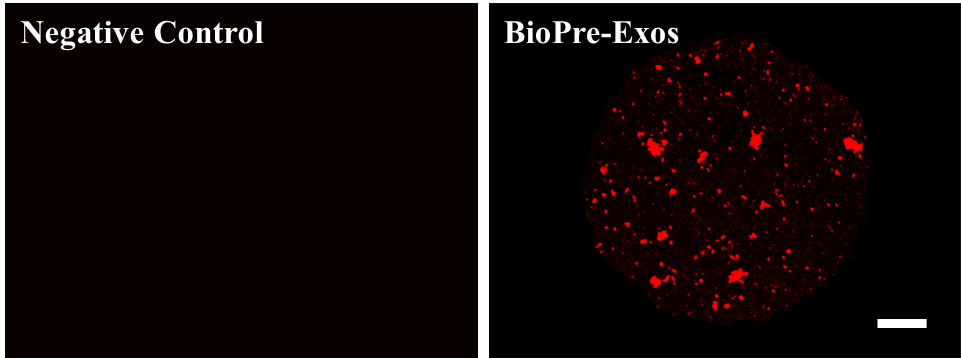


**FIGURE S3** Confocal laser scanning microscopy images of DiD-labeled BioPre-Exos loaded on PDA@GM microspheres.


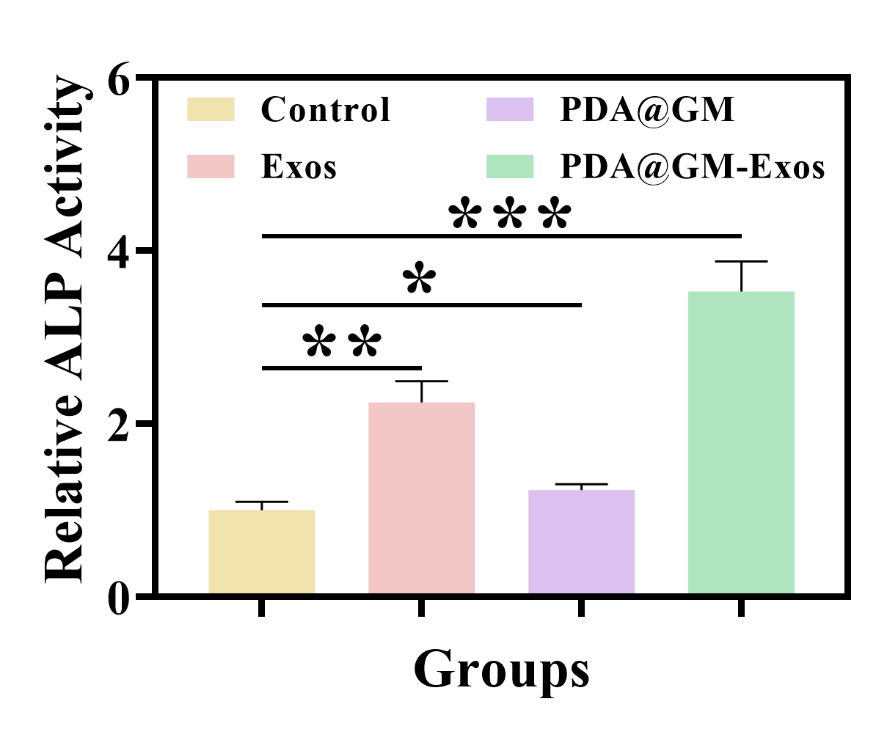


**FIGURE S4** Quantitative analysis of ALP enzymatic activity in BMSCs (n = 3). All data are presented as mean ± SD. Statistical significance was determined using ANOVA with Tukey’s post hoc test. ****p* < 0.001, ***p* < 0.01, **p* < 0.05.
